# Maternal vaginal colonization with selected potential pathogens of neonatal sepsis in the era of antimicrobial resistance, a single center experience from Sri Lanka

**DOI:** 10.1186/s12879-018-3262-y

**Published:** 2018-07-28

**Authors:** Dulmini Nanayakkara, Veranja Liyanapathirana, Chaminda Kandauda, Champika Gihan, Asela Ekanayake, Dinuka Adasooriya

**Affiliations:** 1 Postgraduate Institute of Science, University of Peradeniya, Peradeniya, Sri Lanka; 2 Department of Microbiology, Faculty of Medicine, University of Peradeniya, Peradeniya, Sri Lanka; 3 Department of Obstetrics and Gynaecology, Faculty of Medicine, University of Peradeniya, Peradeniya, Sri Lanka; 4 Faculty of Allied Health Sciences, University of Peradeniya, Peradeniya, Sri Lanka

**Keywords:** Maternal colonization, Sri Lanka, Antimicrobial resistance

## Abstract

**Background:**

Maternal vaginal colonization with antibiotic resistant organisms is a growing concern in countries with high antibiotic resistance rates.

**Methods:**

A low vaginal swab was collected from mothers on admission, on discharge and a peri-rectal swab was collected from the neonates born to these mothers on discharge. Routine microbiological methods were used to identify the colonization rates for *Escherichia coli*, *Klebsiella* spp. and *Streptococcus agalactiae*.

**Results:**

The pre-delivery colonization rate among the 250 participants for total Enterobacteriaceae was 18.8%. The colonization rates for *Klebsiella* spp., *E. coli* and *S. agalactiae* were, 12.4, 5.6 and 14.8% respectively. Two *Klebsiella* spp. and two *E. coli* isolates were confirmed to be exentend spectrum β lactamase (ESBL) producers with the commonest resistant determinant being *bla*_CTX-M_. Post-delivery swabs were collected from 130 participants and the colonization rates were 41.5% for Enterobacteriaceae, 25.4% for *Klebsiella* spp., 10.8% for *E. coli*, and 10.8% for *S. agalacteiae*. Three *Klebsiella* isolates and one *E. coli* isolate were confirmed to be ESBL producers with the commonest resistant determinant being *bla*_CTX-M_. Considering the 130 participants with both samples, there was a significant increase in the colonization with any Enterobacteriaceae and *Klebsiella* spp. (*p* < 0.05). Peri-rectal swabs were collected from neonates in 159 instances. The isolation rates for Enterobacteriaceae was 34%. The genus specific isolation rate for *Klebsiella* was 21.4% while the rates for *E. coli* and *S.agalactiae* were 10.1 and 5.7% respectively. Two of the *E. coli* were confirmed to be ESBL producers while none of the klebsiellae were identified to be so. Considering these 159 instances where both the mother and baby were sampled, random amplification of polymorphic DNA (RAPD) analysis revealed that Enterbacteriaceae with same strain type was present in 6.9% of the instances, indicating possible transfer between the mother and neonate. The transfer rate for ESBL producers were 0.6%.

**Conclusions:**

The lower level of antimicrobial resistance among these potentially community acquired isolates is encouraging. However, in view of the increasing level of resistance reported elsewhere in the region, regular monitoring is warranted.

## Background

Antimicrobial resistance is a major global health issue of the current era. Resistant organisms, initially found in health care environments are now being found commonly in community settings. Asymptomatic colonization with resistant organisms is an area that needs to be studied further as these organisms may subsequently cause serious infections in other sites of the given individual or in susceptible individuals [1–3].

Neonatal sepsis with antibiotic resistant organisms is an emerging issue. Neonatal sepsis associated with antibiotic resistant Gram negatives is a recognized problem in the Indian Subcontinent [4]. While there are different risk factors for early onset neonatal sepsis (EOS), defined as infections occurring within the first 7 days of birth, majority of the organisms causing EOS are colonizers of the maternal birth canal [5, 6]. Therefore studying the resistance pattern among the maternal vaginal colonizers during late pregnancy could be used as a proxy to predict the potential burden of EOS with antibiotic resistant organisms.

Studies on maternal colonization with *Streptococcus agalactiae* are frequently reported while studies on Gram negative colonization are not that common. Colonization rate with extended spectrum β lactamase (ESBL) producing Enterobacteriaecae among pregnant women being admitted for delivery in Madagascar has been found to be 18.5% [7]. The isolation rate for ampicillin resistant *Escherichia coli* was found to increase from 25 to 36% between pre and post-partum periods in a study conducted in Florida, USA while the isolation rates for ampicillin resistant Enterobacteriaciaea was found to increase from 38% in the antenatal period to 51% in the post-partum period [8].

There is an association between bacteria colonizing mothers and their babies [9]. Neonatal colonization with antibiotic resistant organisms also has multiple risk factors. However parental maternal colonization with resistant organisms has been identified as a key risk factor for neonatal colonization in some studies while not in others [10, 11].

Neonatal colonization with antibiotic resistant bacteria may be a transient phenomenon, or it may result in neonatal sepsis. This may also lead to outbreaks associated with resistant organisms [3]. Furthermore, evidence also suggest that those who are colonized may remain carriers for a long period [12].

Despite its obvious importance, research in to the epidemiology of perinatal transmission of resistant organisms remains a neglected area [13]. The current study was conducted with the objectives of identifying the rate of vaginal colonization with multidrug-resistant (MDR) *Klebsiella* spp. and *E. coli* among patients being admitted for term vaginal delivery, identifying the rate of vaginal colonization with Group B streptococci and enterococci among same patients, identifying the rate of peri-rectal colonization with multidrug-resistant *Klebsiella* spp. and *E. coli* among infants born to the same mothers and identifying the rates of potential transfer between mother and baby.

## Methods

This was a prospective observational study which included data gathered using a questionnaire and a laboratory analysis. Subject recruitment and sample collection was conducted at the Professorial Obstetrics Unit, Teaching Hospital, Peradeniya, Sri Lanka and laboratory analysis was carried out in the Antimicrobial Laboratory, Department of Microbiology, Faculty of Medicine, University of Peradeniya, Sri Lanka.

Ethical approval was obtained from the institutional ethical review committee, Faculty of Medicine, University of Peradeniya, Sri Lanka. Informed written consent was obtained from the subjects prior to recruitment to the study.

Mothers being admitted for term vaginal delivery between 01/10/2015 to 06/01/2016 were included in the study while the admission of the baby to special baby care unit, heavy bleeding per-vagina and emergency lower segment Caesarian section (LSCS) were considered as exclusion criteria. Following data were collected from the participants, age of the mother, occupation, number of family members at home, parity, gestation, hospitalizations within the preceding 3 months, history of taking antibiotics within the preceding 3 months, mode of delivery, date of delivery, weight of the baby and date of discharge.

Subjects were selected according to convenience sampling method. Each subject was given a study number and details of the participant, her baby and samples were collected under the given number. Under each subject number, three swabs were collected as,

- A low-vaginal swab from mother collected at the time of admission to antenatal ward,
- A low-vaginal swab from mother collected at the time of discharge from postnatal ward,
- A peri-rectal swab from the baby of the mother collected on discharge.

All swabs were transported to the laboratory in Amie’s transport medium within 2 h of collection. Swabs were stored at − 20 °C till further testing.

All three swabs were inoculated on to quality controlled Blood agar and MacConkey agar plates and low vaginal swabs were then enriched in quality controlled Todd Hewitt broth. All plates and enrichment broth were incubated at 37 °C for 18–24 h. Lactose fermenting colonies of Gram negative bacilli or non-lactose fermenting, oxidase negative colonies of Gram negative bacilli were presumptively selected further identification. Up to ten morphologically distinct colonies were picked from per plate. These were purified and stored at − 80 °C before further identification and sensitivity testing.

Preliminary identification of Gram negative isolates was carried out using the oxidase test, oxidative/fermentative test (O/F Test) and motility test. Identification up to species level was done according to the Cowan and Steel’s Manuel for the Identification of Medical Bacteria including sugar fermentation tests and other biochemical tests. The analysis was restricted mainly to *E. coli* isolates and *Klebiella* species.

Preliminary identification of the Gram positives was carried out with the catalase test, bile esculin test and 6.5% NaCl salt tolerance test. Lancefield grouping of the selected isolates were confirmed by rapid latex agglutination method (Streptococcal Grouping kit, Thermo-Scientific, USA).

Antibiotic Sensitivity Testing (ABST) with disc diffusion method was done on Enterobacteriaceae isolates identified up to species level according to clinical and laboratory standards institute (CLSI) guidelines for cefotaxime, ceftriaxone, ceftazidime, imipenem, ertapenem, gentamicin and ciprofloxacin [14, 15].

Enterococcus isolates were sub cultured on vancomycin resistant enterococci (VRE) agar medium (Thermoscientific, 2016) to screen for vancomycin resistance. Minimum Inhibitory Concentration (MIC) for vancomycin was done on isolates identified as potential VRE using agar dilution method [14, 16].

Zone diameters for cefotaxime and ceftazidime for *E. coli* and *Klebsiella* species were used to identify potential ESBL producers according to the CLSI 2015 guidelines [14]. Those identified as potential ESBL producers were further tested with the combined disk test. All isolates demonstrating non-susceptibility for ertapenem and/or imipenem were tested for carbapenemase production by the Modified Hodge Test (MHT).

*Bla*_CTX-M_, *bla*_SHV_ and *bla*_TEM_ were looked for as genetic determinants of ESBL production by a previously described multiplex PCR [17]. In instances where the same Enterobacteriaceae with the same antibiograme were isolated from the mother and the neonate, these isolates were selected for typing with random amplification of polymorphic DNA (RAPD). RAPD was performed using previously described primers and PCR conditions [18]. Dendrogrammes were drawn using the JelJ software using the Unweighted Pair Group Method with Arithmetic Mean (UPGMA) method [19]. Isolates that had a similarity of > 75% in the Dice index at a tolerance of 3.5 were considered as possibly having the same origin indicating potential transmission between the mother and baby. Enterobacteriaceae isolates that were non-susceptible for ≥3 antimicrobial categories as defined by Magiorakos et al. were identified as being MDR isolates [20].

## Results

During the study period, 250 term pregnant women who were admitted to the study unit, were recruited for the study. On admission low-vaginal swabs (swab a) were collected from all the participants (250). Pre-delivery colonization rates were calculated including all 250 participants recruited.

Post-delivery swabs (swab b) were collected from 130 of the 250 participants. The 120 participants in whom a post-delivery swab was not available belonged to one of the following four categories; delivered via emergency LSCS (*n* = 56), discharged without delivery (*n* = 19), admission of the baby to special care baby units (*n* = 8) or discharged prior to research team collecting the swab (*n* = 37).

**Fig. 1:**
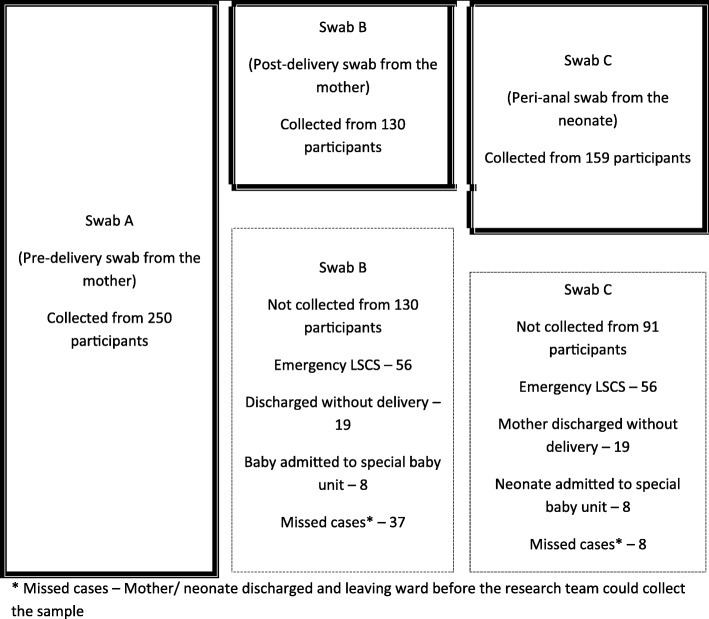
Patient recruitment

A peri-rectal swab was collected from 159 babies (swab c). All three swabs were collected from 120 participant mother-baby combinations while a maternal and a neonatal swab (a and/or b + c) was available in 159 participant pairs (Fig. 1). Differences in pre and post-delivery colonization rates were calculated only for the 130 participants where both swabs (a and b) were available. Potential mother to child transmissions were assessed in instance where at least one maternal swab and the peri-anal swab from the baby were available (*n* = 159).

Maternal ages ranged from 17 to 41 years with a mean age of 28.3 (SD 5.3 years). Majority of the mothers were in their first pregnancy (110/250, 44%). Hundred and eighty nine (75.6%) of the participants were housewives while the others were employed. Twenty five (10%) participants had one or more co-morbid conditions that were pre-existing or pregnancy associated. Fifteen of the participants had either gestational or non-gestational diabetes mellitus, 8 participants had gestational or essential hypertension, 2 participants were on treatment for hypothyroidism and 1 participant was diagnosed to have anaemia. The mean duration of hospitalization was 3.9 days (SD 2.1), the mean stay till delivery was 1.8 days (SD 1.9) and the mean duration of hospital stay for the baby was 2.2 days (SD 1.2).

### Pre-delivery bacterial colonization (*N* = 250)

*S. agalactiae* colonization rate was 14.8% (*n* = 37) while the colonization rate for *Enterococcus* spp. was 24.8% (*n* = 62). All enterococcus isolates had vancomycin of MICs < 2 μg/ml.

The colonization rate for at least one Enterobacteriaceae isolate was 18.8% (*n* = 47). The genus specific colonization rate for *Klebsiella* spp. was 12.4% (*n* = 31). Two *Klebsiella* spp. isolates (2/31,6.45%) were confirmed to be ESBL producers harbouring all three genetic determinants tested (*bla*_CTX-M_, *bla*_SHV_ and *bla*_*TEM*_). All *Klebsiella* spp. were sensitive to imipenem, meropenem and ertapenem as well as ciprofloxacin and levofloxacin. Only one isolate could be defined as a MDR organism.

The species specific colonization rate for *E. coli* was 5.6% (*n* = 14). Two *E. coli* isolates were confirmed to be ESBL producers (2/14, 14.3%) and harboured *bla*_CTX-M_ only. All *E. coli* isolates were sensitive to imipenem, meropenem and ertapenem. All *E. coli* isolates were also sensitive to ciprofloxacin and levofloxacin. None of the *E. coli* were MDR organisms.

Pre-delivery colonization rate for ESBL producing *Klebsiella* spp. or *E.coli* was 1.6% (4/250). Carbapenem resistance rate for *Klebsiella* spp. or *E.coli* was 0%. The pre-delivery colonization rate for MDR *Klebsiella* spp. or *E.coli* was 0.4% (1/250).

### Post-delivery colonization rates (*N* = 130)

Hundred and thirty participants were sampled in the post delivery period. *S. agalactiae* colonization rate was 10.8% (*n* = 14) while the colonization rate for *Enterococcus* spp. was 17.7% (*n* = 23). All enterococcus isolates had vancomycin of MICs < 2 μg/ml.

Fifty four patients (41.5%) were colonized with at least one Enterobacteriaceae. Thirty three (25.4%) patients carried *Klebsiella* spp. Three isolates (3/33, 9.1%) were confirmed to be ESBL producers. One of the ESBL producers carried all three determinants of ESBL, namely *bla*_CTX-M_, *bla*_SHV_ and *bla*_TEM,_ while one isolate each carried *bla*_CTX-M_, and *bla*_TEM.._ One isolate was found to be resistant to ertapenem and imipenem while being sensitive to meropenem. This isolate was found to be a carbapanamase producer by the MHT. Two isolates were resistant to ciprofloxacin and levofloxacin (2/33,6.1%). Only one isolate could be defined as a MDR organism for being resistant to more than three classes of antibiotics.

The species specific colonization rate for *E. coli* was 10.8% (*n* = 14). One isolate was confirmed to be an ESBL producer harbouring both *bla*_CTX-M_ and *bla*_TEM._ All isolates were sensitive to meropenem, imipenem, ertapenem, ciprofloxacin and levofloxacin. None of the *E. coli* were MDRs.

Therefore, the post-delivery colonization rate with ESBL producing *Klebsiella* spp. or *E. coli* was 3.1% (4/130). Colonization rate for carbapenem resistant *Klebsiella* spp. or *E. coli* was 0.8% (1/130). The colonization rate for MDR *Klebsiella* spp. or *E. coli* among the post-delivery cohort was also 0.8% (1/130).

### Comparison of pre and post-delivery colonization rates

This analysis was restricted to the 130 instance where a pre and a post-delivery swab were taken from the same patient. There was a significant increase in the colonization with any Enterobacteriaceae and *Klebsiella* spp. (Table 1). The reduction in the colonization rates for *Enterococcus* species was also significant. Changes seen in *S. agalactiae* and *E. coli* were not statistically significant.

**Table 1:** Comparison of colonization among pre and post delivery periods (*n* = 130)

Organism	Colonization rate on admission	Colonization rate on discharge	Significance
*S. agalactiae*	21 (16.2%)	14 (10.85)	0.241 (Fishers Exact)
*Enterococcus* spp	30 (23.1%)	23 (17.7%)	0.015 (Chi square)
Enterobacteriaceae^a^	31 (23.8%)	54 (41.5%)	0.013 (Chi square)
*E. coli*	8 (6.25%)	14 (10.8%)	0.598 (Fishers Exact)
*Klebsiella* spp	23 (17.7%)	33 (25.4%)	0.036 (Chi square)

^a^Differences in rates were not calculated for individual Enterobacteriaceae other than *E. coli* and *Klebsiella* spp. due to low numbers

Considering antibiotic resistance, the MDR *Klebsiella* spp. or *E. coli* rate in the 130 patients where a both swabs were available was 0.8% (1/130) in the pre-delivery period as well as the post delivery period. The colonization rates for ESBL producing *Klebsiella* spp. or *E. coli* were 2.3% (3/130) and 3.1% (4/130) during the pre and post delivery periods. The difference was not statistically significant.

### Neonatal colonization (*N* = 159)

*S. agalactiae* colonization rate was 5.7% (*n* = 9) while the colonization rate for *Enterococcus* spp. was 11.9% (*n* = 19). MIC of one enterococcus for vancomycin was 4 μg/ml while it was < 2 μg/ml in the other isolates. Enterobacteriaceae were found in 34.0% (*n* = 54) of neonates while *Klebsiella* spp. were found among 21.4% (*n* = 34) and *E. coli* was found in 10.1% (*n* = 16) of the babies. Two *E. coli* isolates were confirmed to be ESBL producers while none of the *Klebsiella* species were found to be ESBL producers. The two ESBL producers harboured both *bla*_CTX-M_, and *bla*_TEM._ None of the *Klebsiella* spp. or *E. coli* isolates were resistant to carbapenems, ciprofloxacin or levofloxacin. None of the isolates were MDRs. Therefore, the colonization rate among the neonates with ESBL producing *Klebsiella* spp. or *E. coli* was 1.2% (2/159) and the rates were 0% for carbapenem resistant or MDR *Klebsiella* spp. or *E. coli.*

### Potential transfer of organisms from mother to baby (*N* = 159)

This was assessed using the 159 pairs of maternal-neonatal pairss where either pre and/or post delivery swab from the mother and the neonatal peri-rectal swab was available. *S. agalactiae* was found in both the mother and the baby in four instances (2.5%). While *Enterococcus* species was found in mother –baby pairs in 13 instances (8.2%).

There were 23 (14.5%) instances where both the mother and the baby were colonized with the same Enterobacteriaceae with the same antibiogramme, where 4 (2.5%) maternal-neonatal paires were colonized with *E. coli*, 18 (11.3%) were with *Klebsiella* spp. (*K. oxytoca −* 3, *K. pneumoniae* – 15) and one was with *Enterobacter cloacae* (*n* = 1).

**Fig. 2:**
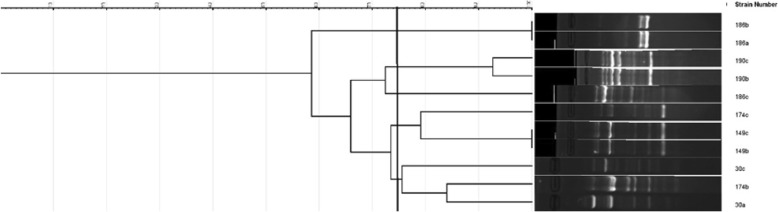
Dendrogramme to demonstrate potential transfer from mother to baby – *Escherichia coli* isolates. Numbers along the axis indicate the percentage similarity in Dice index. Isolates demonstrating similarity > 75%: 190b – 190c and 149b – 149 c. a: pre-delivery low vaginal swab, b: post-delivery low vaginal swab, c: peri-rectal swab from the baby. The number denotes the study number allocated to a given mother-baby pair

**Fig. 3:**
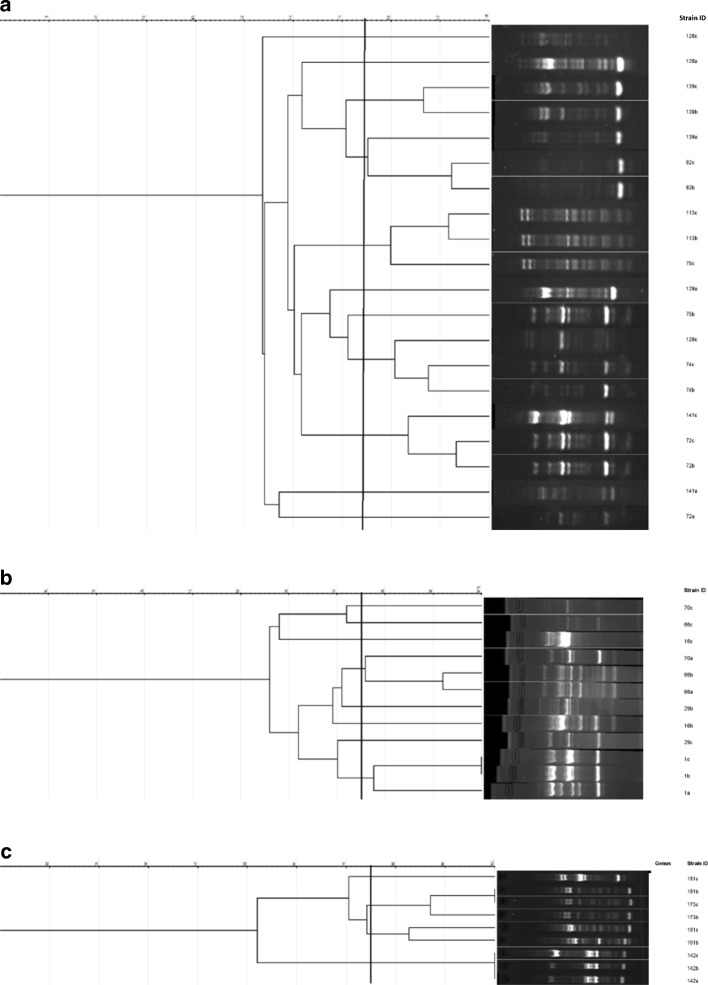
Dendrogramme to demonstrate potential transfer from mother to baby – *Klebsiella* isolates. Numbers along the axis indicate the percentage similarity in Dice index. Isolates demonstrating similarity > 75%: 1b-1c, 72b-72c, 74b-74c, 82b-82c, 113b-113c, 139b-139c, 142a-142b-142c, 173b-173c. a: pre-delivery low vaginal swab, b: post-delivery low vaginal swab, c: peri-rectal swab from the baby. The number denotes the study number allocated to a given mother-baby pair. *We could not compare all *Klebsiella* isolates in a single gel due to inconsistencies of the marker used. Therefore, the three gels used were analysed separately as (**a**,**b** and **c**). However, isolates from a single mother-neonate pair were in the same gel

Analysis of RAPD results revealed that two of the four *E. coli* found in maternal neonatal pairs showed similarity of more than 75% in at a tolerance of 3.5 (Fig. 2). Of the 18 pairs of *Klebsiella* isolates, eight pairs showed similarity of more than 75% in at a tolerance of 3.5 (Fig. 3). The *Enterobacter cloacae* found in the maternal-neonatal pair showed a similarity of > 75% (Not shown in figure). Therefore, considering only the isolates with > 75% similarity in Dice index, eleven pairs isolates (11/159, 6.9%) could have been from the same origin therefore, potentially transferred between the mother and the baby. Only one (1/159, 0.6%) of these 11 was an ESBL producer and while none were MDR organisms.

## Discussion

We identified 47 expectant mothers to be colonized with Enterobacteriaceae giving a percentage of 18.8% while the genus specific colonization rate for *Klebsiella* spp. was 12.4% (*n* = 31) and the species specific colonization rate for *E. coli* was 5.6% (*n* = 14).

In a study conducted in Pakistan in 2008, the *E. coli* colonization among pre-delivery mothers has been reported as 13.7% (*n* = 100), while the colonization rate for *Klebsiella pneumoniae* was 10.5% (*n* = 77) and for β haemolytic streptococci it was 8.8% (*n* = 64) among 1923 subjects [21]. A study conducted in 2012 in Argentina revealed an 18.55% (*n* = 48) colonization rate for Enterobacteriaceae among 259 pregnant women, and the species specific colonization rates were 14.3% (*n* = 37) for *E.coli* and 1.2% (n = 3) *Klebsiella pneumoniae* [22]. *E. coli* colonization percentage in pregnant women in Assam, India during 2014 was reported as 16.26% among 246-screened samples [23]. Our overall colonization rate for Enterobacteriaecae was similar to the Argentinean study. Our colonization rate for *E .coli* at 5.6% was lower than that reported from the neighboring India and Pakistan. Our colonization rate for *Klebsiella* spp. at 12.4% was similar to that reported from Pakistan. These variations might reflect differences in sample collecting, processing or hygienic and environmental conditions in the given locales.

In the current study, we found a pre-delivery colonization rate of *S. agalactieae* was 14.8% (*n* = 37) while the colonization rate for *Enterococcus* spp. was 24.8% (*n* = 62). Colonization rate for *S. agalactieae* reported by Dissanayake et al. in the same study unit was 24% [24]. The difference may be due to the direct enrichment employed Dissanayake et al. whereas in this study, enrichment for GBS was done following initial plating of the swabs. A temporal shift could also explain the given difference as there has been a 5 year lapse between the studies.

We have identified a significant increase in the colonization rate with Enterobacteriaceae during the post-delivery period. This has been the case in previous studies for over decades, and the presence of altered blood and dead tissue in the vaginal tract during this period with the associated changes in the environment has been cited as the possible reasons [25].

The colonization rates for ESBL producing *Klebsiella* spp. or *E .coli* were 1.6, 3.1 and 1.2% among the pre-delivery, post-delivery and the neonatal periods. Considering the 130 participants where both pre and post delivery samples were available, the rates did not differ significantly. The rates identified are much lower than that reported from Madagascar and other areas for the pre-delivery colonization rates [7]. In Sri Lanka, hospital isolates have been reported to be much higher [26, 27]. However, the previous Sri Lankan studies were conducted considering patients who had clinical illnesses, such as urinary tract infections. Even among patients with community onset infections, the exposure to previous antibiotics has been high [27]. However, our study cohort was pregnant females, who have minimal health care exposure in hospital settings. Only 10% of the participants had any co-morbid conditions, this is also favourable for a lower rate of colonization with resistant organisms [28]. It is encouraging to see that despite high resistance rates among hospital isolates, the rates remain lower normal flora of healthy groups. Furthermore, the length of stay in hospital among the study cohort was relatively short (Mean, 3.9 days), further contributing to the lower ESBL colonization rate, even among the post-delivery period.

The colonization rates for MDR *Klebsiella* spp. or *E. coli* were 0.4, 0.8 and 0% among the pre-delivery, post-delivery and the neonatal periods. Considering the 130 participants where both pre and post delivery samples were available, the rates did not differ significantly. The explanations given for the lower ESBL colonization rates are valid for the lower rates of colonization with multi-drug resistant organisms as well.

Among the ESBL producing Enterobacteriaceae, the commonest genetic determinant of resistance was found to be *bla*_CTX-M_, keeping up with previous studies including more community onset samples and unlike those originating from health care associated samples [26, 29]..

The potential maternal-neonatal transfer rate identified in the study was 6.9% for Enterobacteriaceae and it was 0.6% for ESBL producers. While the rates are not too high, keeping a vigilant eye on changing epidemiology is needed.

As the transfer events for MDR organisms was low, we did not perform a risk factor analysis.

The potential for the different colonizers identified in this study to cause EOS differs and mortality associated with EOS caused by different organisms also differ [30]. Furthermore, there is scarcity of data on organisms causing EOS in Sri Lanka. Therefore, what we have identified here, as colonization rates among mothers and neonates who are healthy, can only be used as a surrogate to the pool of organisms that may emerge as causing EOS. Furthermore, we excluded the babies who were admitted to the special care baby units from the analysis due to logistic reasons. A follow-up study where infants are monitored to see if they develop subsequent neonatal sepsis and if so correlate the causative organisms for neonatal sepsis with the colonizers identified from the mother and baby would be useful to establish the actual risk of antibiotic resistant maternal colonizers causing sepsis.

There are a few limitations to this study. We collected a low vaginal swab from the mother and a peri-rectal swab from the child while a peri-rectal swab from the mother may have helped to identify more women colonized with resistant organisms [31]. Samples were stored in – 20 °C prior to testing, which is not the ideal but the only feasible method given the circumstances. We identified the resistance rates only among *Klebsiella* spp. and *E. coli*. Not all Enterobacteriaceae or Gram negatives were considered. We also did not perform sensitivity testing on the Group B streptococci. The sample size included was also relatively lower. We gathered patient details including co morbidities by direct questioning of the participant than by going through hospital records, which may not be the most accurate method to gather the data. As we had to change the molecular marker used for agarose gels in during the study period, we could not compare the potential transfer of all *Klebsiella* isolates in a single dendrogramme. However, as what we were interested in is to compare the nature of isolates obtained from a single maternal-neonate pair than the whole cohort, this did not affect the results.

Despite the limitations, our study is one of the very first in Sri Lanka which looks at the potential colonization with resistant organisms among a healthy population.

## Conclusions

The lower rates of resistance identified among vaginal colonizers from pregnant females being admitted for delivery including ESBL producers, MDR organisms and carbapenem resistant isolates is encouraging.

However, steps need to be taken to periodically monitor this group of individuals as an increase resistance rates among vaginal colonizers in term pregnant women would have implications on the treatment options used for puerperal sepsis as well as early onset neonatal sepsis.

## Abbreviations

CLSI: Clinical and Laboratory Standards Institute
EOS: Early onset neonatal sepsis
ESBL: Extended spectrum βlactamase
GBS: Group B streptococci
MDR: Multi drug resistant
MHT: Modified Hodge test
MIC: Minimum inhibitory concentration
PCR: Polymerase chain reaction
RAPD: Random amplification of polymorphic DNA
SD: Standard deviation
UPMGA: Unweighted Pair Group Method with Arithmetic Mean
VRE: Vancomycin resistant enterococci
